# Dynamic consent: a royal road to research consent?

**DOI:** 10.1136/jme-2024-110153

**Published:** 2024-07-24

**Authors:** Andreas Bruns, Eva C Winkler

**Affiliations:** 1 Section of Translational Medical Ethics, National Center for Tumor Diseases, Department of Medical Oncology, University Hospital Heidelberg, Heidelberg, Baden-Württemberg, Germany

**Keywords:** Autonomy, Informed Consent, Ethics, Ethics- Medical, Philosophy

## Abstract

In recent years, the principle of informed consent has come under significant pressure with the rise of biobanks and data infrastructures for medical research. Study-specific consent is unfeasible in the context of biobank and data infrastructure research; and while broad consent facilitates research, it has been criticised as being insufficient to secure a truly informed consent. Dynamic consent has been promoted as a promising alternative approach that could help patients and research participants regain control over the use of their biospecimen and health data in medical research. Critical voices have focused mainly on concerns around its implementation; but little has been said about the argument that dynamic consent is morally superior to broad consent as a way to respect people’s individual autonomy. In this paper, we identify two versions of this argument—an information-focused version and a control-focused version—and then argue that both fail to establish the moral superiority of dynamic over broad consent. In particular, we argue that since autonomous choices are a certain species of choices, it is neither obvious that dynamic consent would meaningfully enhance people’s autonomy, nor that it is morally justifiable to act on every kind of consent choice enabled by dynamic consent.

## Introduction

 Informed consent is a central principle of medical research ethics. But with the rise of biobanks and data infrastructures for medical research, the principle of informed consent has once again become the focus of substantial discussion.^1^ It has become possible to store large amounts of biospecimen and digitalised health data from human donors and to make them available to researchers around the globe for research uses beyond the initial purpose of collection. While further usability is a central demand of state-of-the-art medical research, obtaining informed consent from donors proves challenging in the face of the unpredictability of future research uses and the sheer amount of possibly useful future applications of stored resources.

As an answer to this challenge, the concept of ‘broad consent’ has been conceived to replace the traditional standard of specific consent for biobank and data infrastructure research. In the broad consent model, donors are asked to provide their consent to kinds of future research uses instead of consenting to each use specifically. However, while this facilitates research, some have argued that broad consent fails to constitute sufficiently informed consent or that it would not provide the level of control necessary for authentic self-determination.^2 3^ Such criticism has fostered the emergence of alternative consent models, of which ‘dynamic consent’ has received special attention. In the dynamic consent model, donors first agree that their biospecimen or data may be included in some kind of research platform and then receive regular information about new studies coupled with requests to accept or reject those research uses.^4–7^ In principle, dynamic consent thus aims to enable study-specific consent for biobank and data infrastructure research.

Its supporters present dynamic consent as a way to help donors regain control over their contributions to medical research, emphasising its potential to conceptualise the research process as a shared social activity, a ‘social agreement between researchers and research participants’ (p. 7),^5^ with donors being placed ‘at the centre of decision making as equal partners in the research process’ (p. 372).^8^ Critical voices have focused mainly on challenges around implementation, increased demands on consent management, problems with the reliance on digital technologies and the risk of ‘consent fatigue’ on donors’ part.^9–11^ But little has been said about the underlying ethical argument that dynamic consent would be morally superior to broad consent as a way to respect the autonomy of donors (the ‘autonomy argument’).

In this paper, we first identify two versions of the autonomy argument—an information-focused version and a control-focused version—and then raise doubts that they can establish the moral superiority of dynamic over broad consent. The information-focused autonomy argument is insufficient to justify a study-specific consent; the control-focused autonomy argument is based on the false assumption that respect for autonomy would require respect for all choices, regardless of their nature or appreciability. We conclude that while dynamic consent interfaces can, under certain conditions, provide a useful technology to address the practical and informational challenges around broad consent, there are decisive reasons against the use of these interfaces as a means to enable study-specific consent for biobank and data infrastructure research.

## The many (inter)faces of dynamic consent

The term ‘dynamic consent’ is often used to refer to digital interfaces that enable continuous communication between researchers and participants, and that help manage the disclosure of information and provision of consent. While there is a great deal of diversity in the possible features and applications of these interfaces, their declared common goal is to establish ‘dynamic participant-researcher partnerships’ (p. 1305).^12^ In general, dynamic consent interfaces are designed to help participants personalise their consent and researchers to satisfy the relevant legal and ethical requirements regarding the use of human biospecimen and health data.

However, there is a difference between consent technologies and consent models; and while the flexibility inherent to dynamic consent interfaces is often taken to be a major advantage, proponents of dynamic consent are not always clear on the precise claim they are advancing. Sometimes, the claim might just be that researchers should use digital interfaces to support the broad consent process, as these can make it easier to update participants about ongoing research activities and to re-obtain consent, if necessary. However, at least sometimes, its proponents seem to think of dynamic consent as a distinct consent model, one that should replace broad consent.^4–8^ We therefore begin by asking what separates the dynamic consent model from broad consent and what reasons we have, according to its supporters, to prefer the dynamic to the broad consent model.

Medical research with human biospecimen or health data does not require the direct involvement of human research subjects. While this means that this type of research carries no direct risks to the health or well-being of those contributing to it, it comes with other kinds of risks or concerns. In particular, it is possible to re-identify donors if biospecimen or data are not properly anonymised or cannot be made fully anonymous, as is the case with genetic and genomic data, for instance.^13^ Moreover, the increasing amounts of data available can make it possible to indirectly identify a person by linking health data to other kinds of data available elsewhere. Biobank and data infrastructure research, although not ‘interventional’ in the traditional sense, is therefore associated with especially informational risks to donors.^1 13^

This is often taken to have two normative implications. First, resources originating from human subjects should be de-identified (as appropriate to the type of resource), securely stored and protected from unauthorised access in order to prevent that people suffer any harms or disadvantages as a result of their contribution to medical research. Second, people should decide for themselves, after being adequately informed, whether or not they are willing to provide their biospecimen or health data for medical research. Thus, the requirement to seek consent from patients and research subjects is usually thought to extend to the use of their biospecimen and health data. For example, the Declaration of Helsinki states that for ‘medical research using identifiable human material or data… physicians must seek informed consent for its collection, storage and/or reuse’ (§32).^14^

However, there has been significant uncertainty about what the appropriate model of consent for biobank and data infrastructure research should be. The traditional standard of specific consent (consent to an individual, narrowly defined healthcare intervention or research activity) is unfeasible in the context of state-of-the-art medical research, which unfolds its full potential only where biospecimen and health data can be shared and reused for new (and at the time of consent unforeseeable) research studies without having to seek a new consent from donors each time. Broad consent is meant to facilitate medical research, seeking donors’ one-time consent to a range of future research uses and allowing them to withdraw consent at any time, thereby rejecting any further use.^15^ Sometimes, it might be necessary to re-obtain consent in case that new research uses come up that would fall outside of the scope of the original consent. In contrast to specific consent, however, broad consent shifts the object of consent from individual research activities to kinds of research activities or to a ‘framework for future research of certain types’ (p. 897).^9^

One obvious ethical concern about broad consent is that it might run the risk of becoming an ‘ethically dubious practice’ (p. 521).^16^ It seems that researchers could intentionally make use of people’s ignorance towards the details of planned research activities to obtain consent for activities for which they could not otherwise have hoped to obtain consent. Proponents of broad consent see this as a decisive reason to instal ethical governance and control measures to ensure that the broadness of consent is not abused, and that research aligns with the conditions and limits specified in the original consent. Yet critics of broad consent do not think this to be sufficient. They have raised another concern, namely that broad consent would not sufficiently respect the autonomy of donors. In medical ethics, the notion of autonomy is most commonly used to refer to the autonomy of choices, sometimes called ‘individual autonomy’.^17^ In this regard, informed consent can be understood as a kind of autonomous authorisation of a medical interventions or research activities.^18^ But broad consent, to some, seems inadequate to enable a truly informed and autonomous authorisation.^2–4^ Critics have therefore suggested alternative models of consent, including ‘meta consent’ and ‘dynamic consent’.

Meta consent is the idea ‘that people should be asked how and when they would like to be presented with a request for consent’ (p. 724).^19^ People may, for instance, choose to give their broad consent to some but want to be asked for study-specific consent to other kinds of research activities. Meta consent might initially seem attractive as a compromise between study-specific and broad consent; but it raises a range of other concerns.^20^ Not the least of these concerns is that, if meta consent is meant to overcome the supposed shortcomings of broad consent, then it may not include broad consent as an option. We will not discuss the meta consent model in any more detail here. But our arguments against dynamic consent will equally target the idea that researchers should use meta consent interfaces to seek study-specific consent for biobank and data infrastructure research.

The dynamic consent model, as we understand it, is more stringent here. It is similar to the meta consent model but goes one step further in fully rejecting broad consent.^20^ While broad consent is one of several options in the meta consent model, dynamic consent is based on the premise that, in principle, researchers should seek donors’ consent for each new study. The dynamic consent model, so understood, aims to fully operationalise study-specific consent in biobank and data infrastructure research. This is why Wiertz and Boldt also call the model ‘dynamic specific consent’.^11^ It can be understood as an alternative both to traditional specific consent (which is unfeasible in the context of biobank and data infrastructure research) and to broad consent (which some have felt does not enable autonomous consent). In practice, the lines between broad, meta and dynamic consent may be blurrier than our presentation suggests.^21–23^ But whenever in the following we speak of ‘dynamic consent’, we mean the consent model that aims to operationalise study-specific consent in biobank and data infrastructure research. We speak of dynamic consent ‘interfaces’ to distinguish the technology that can be used to implement dynamic consent from the consent model.

What seems to be the underlying premise of much of the current discussion around dynamic consent is that, notwithstanding practical difficulties, it would be morally ideal if donors could provide dynamic, study-specific consent to biobank and data infrastructure research. This is so because, unlike broad consent, dynamic consent would seem to enable truly autonomous consent. There are two ways to understand the autonomy argument in favour of dynamic consent, first as an argument about information and second as an argument about control. We begin with the information-focused autonomy argument.

## The information-focused autonomy argument

In the dynamic consent model, donors receive more information about the intended uses of their biospecimen and data than in the broad consent model. Of course, this alone need not trouble supporters of broad consent because it does not entail that there is any problem with providing less information. If people are sufficiently informed under broad consent, then refraining from burdening donors with more information in the consent process is rather a good thing. Practice teaches us that consent information materials tend to be too long and would require reduction rather than extension.^24^ This is why proponents of dynamic consent usually go one step further and deny that broad consent is sufficient to allow people to be adequately informed. Broad consent, they say, cannot ‘meet the demands of meaningfully informed consent’ (p. 141).^4^

On the face of it, this is a curious kind of argument. If taken seriously, the argument seems to imply that we may not accept people’s broad consent, even if they wish to provide it. Informedness is a necessary condition for valid consent and if someone’s consent is insufficiently informed (by some standard of what counts as ‘sufficient’ in the relevant context), then it cannot be permissible to accept their consent. However, some donors might wish to support medical research broadly, trusting that their biospecimen and data will be handled with care and used in ways that reflect common moral and political values. They might want to provide their broad consent. In fact, a recent study with German patients with cancer found that most participants preferred broad consent to study-specific consent which showed the lowest acceptance rate among consent models.^25^ But if we take the argument seriously that broad consent cannot be sufficiently informed, then the notion of ‘informed broad consent’ would have to be considered ‘a contradiction in terms’ (p. 897).^9^ Donors would have to be required to provide study-specific consent through a dynamic consent interface. As Johnsson and Eriksson note, this would seem to undermine rather than respect donor autonomy:

To burden participants with ‘information’ that they have not asked for is not to respect their autonomy, but to violate their integrity in the name of empowerment. Forcing people to nurture their authority may be an acceptable (or even commendable) practice in some arenas of life. What we question is whether medical research is such an arena (p. 477).^10^

Taking the information-focused autonomy argument seriously would seem to commit proponents of dynamic consent to the view that, for donors, dynamic consent is not an offer; it is an obligation.

Can defenders of dynamic consent avoid committing to this view? Perhaps, they would want to argue instead that broad consent is insufficient in some other sense. Some people might wish to provide their broad consent to research but others, in turn, might want to know more about how their biospecimen or data will be used specifically before they are ready to give consent. In short, people have diverging informational needs. Dynamic consent could be said to be designed with those in mind who would feel like their informational needs are not taken seriously under broad consent.^7 8^ But again, the problem is how to get from this to the idea that consent needs to be dynamic (if ‘dynamic’ here means study-specific). The fact of diverging informational needs, other things being equal, gives supporters of dynamic consent a reason for wanting to include both broad and study-specific consent (they would be arguing for meta consent). But the fact that some people might want to be asked for study-specific consent cannot justify excluding the option for broad consent.

There is another problem with the argument that dynamic consent is needed because broad consent cannot be sufficiently informed. As we have argued earlier, dynamic consent is intelligible as a distinct consent model only if it aims to operationalise a study-specific consent. Otherwise, dynamic consent interfaces are just possibly useful tools to enable broad or meta consent, which is not what proponents of dynamic consent seem to have in mind. But the idea that donors should be allowed to consent to each study specifically is distinct from the idea that donors should be informed about each specific study. Even if it could be convincingly argued that dynamic consent is superior to broad consent as a way to enable informed consent, it would not follow from this that we should opt for dynamic, study-specific consent. All that could be inferred from this is that we should use dynamic consent interfaces to inform donors about each new study. In principle, broad consent is compatible with this. It is compatible with the claim that donors should be informed about the use—even each use—of their biospecimen and data. In the broad consent model, donors can withdraw consent at any time and thus could decide on the basis of that information if they wish to withdraw consent. There is no need for consent to be study-specific if all we want is for donors to know which studies use their biospecimen or health data; such a dynamically informed, broad consent would be enough.

## Is there an informational deficit?

The question remains whether there is in fact an informational deficit in broad consent and, if so, whether this deficit could be addressed by using dynamic consent interfaces to provide donors with study-specific information, if they wish to receive them. Is it desirable to move from standard broad consent to a dynamically informed one?

To begin with, what precisely is it that critics of broad consent think makes the model deficient? The claim here cannot just be that broad consent is insufficient because, under broad consent, people do not usually know which specific studies use their biospecimen or data because this would amount to the empty claim that broad consent is insufficient because it is broad. But this seems to be the underlying assumption of much of the criticism against broad consent. For instance, Kaye *et al*, after stating that broad consent cannot meet the demands of meaningfully informed consent, continue:

As the nature of biomedical research changes, the social contract between participants and researchers needs to evolve with it. If biobank research is open-ended and ongoing then information technologies offer the possibility for participant involvement similarly to extend through time. Individuals need no longer be passive human ‘subjects’ but can be engaged over time and recognised as active, interested and valued research participants (p. 142).^4^

While recognising donors as active participants rather than passive subjects sounds like a valuable aim, no argument is provided for the claim that broad consent cannot be sufficiently informed. To establish this, it is not enough to say that because biobank and data infrastructure research are ‘ongoing’, consent needs to be ‘ongoing’ as well; more needs to be said.

Perhaps, the criticism against broad consent aims at the concern that, under broad consent, we can never be certain that people would have agreed to a specific use that they have not known about. Thus, it cannot be guaranteed that when specific research uses come to be known to donors, none of them will object that they did not expect this kind of use to be covered by their consent.^10^ However, this should be a concern only where the relevant research use falls outside of the scope of the original consent, in which case it would usually be required to seek re-consent anyway. Researchers can do their best to ensure that donors understand that if they agree to certain kinds of research, then this implies that their biospecimen and data may be used broadly for these kinds of research. From time to time, they might face situations in which it is not obvious whether a research use falls within the scope of the original consent. But apart from that, researchers and donors alike must be able to depend on the reliability of the consent transaction.

Moreover, it should not be taken for granted that providing study-specific information to donors would solve the above problem. It has rightly been pointed out that more information ‘in itself does not necessarily make a consent more informed’ (p. 899).^9^ This is so because there is a difference between the disclosure and understanding of information.^26 27^ And while it is clear that dynamic consent would help facilitate the disclosure of information, it is less obvious that this would significantly contribute to how well people understand the implications of research. Empirical studies have shown that people participating in clinical trials or research studies often cannot report back information that has been disclosed to them prior to participation, including information on the objectives and associated risks of research.^28–30^ Even when it comes to study-specific consent, it is often an open question whether disclosed information is properly received and understood.

While this should not lead researchers to think that trying to inform people about the nature, aims and risks of medical research is a lost cause, it suggests that introducing further ways to provide people with more information, in itself, is insufficient to promote understanding. Critics of dynamic consent have raised concerns that increasing the frequency and amount of information disclosed might even obstruct understanding by making it harder to distinguish between relevant and irrelevant information.^9 10^ Moreover, the opportunity to deliver information in large amounts or great detail might, to some extent, seem to relieve researchers from the duty to carefully consider what kind of information should be disclosed and how. Consent can never be fully informed; what matters is that it is relevantly informed.^17^ Perhaps, there is no harm in using dynamic consent interfaces to provide donors with more information about specific studies, if this helps donors better understand what it is that they are involved with when providing broad consent to biobank and data infrastructure research. But for this strategy to be successful, more needs to be said about how these interfaces should be used. Otherwise, dynamic consent interfaces run the risk of becoming mere informational dumping grounds.

In summary, if dynamic consent is meant to be an offer to those who would want to know more about the details of biobank and data infrastructure research, then there is no reason to opt for study-specific consent. In general, it seems best practice for biobanks and data infrastructures, regardless of the consent model they use, to maintain information sites that are easily accessible and regularly updated, and that inform people (not just donors but the broader public) about research studies, projects and initiatives. In such an environment, it is unclear why we should think that broad consent would not enable an adequately informed consent. Consent interfaces that enable a continuous and potentially unlimited provision of information cannot relieve researchers from the obligation to carefully select information according to relevance and to present that information in ways that promote understanding on the donors’ part. And the diverging informational needs of donors might sometimes better be served by making additional information available on separate sites, rather than in an increasingly complex consent process. If used appropriately, however, there is no reason why dynamic consent interfaces cannot provide a useful tool to support the information and consent processes central to biobank and data infrastructure research, making it easier for researchers to keep in touch with donors and for donors to exercise their right to withdraw consent or provide re-consent, if needed.

## The control-focused autonomy argument

In order to justify the dynamic consent model (as dynamic, study-specific consent), its proponents therefore cannot just rely on the claim that dynamic consent would be superior to broad consent as a way to enable truly informed consent. They need to introduce the idea that broad consent is insufficient because it does not give donors enough control over their biospecimen or health data. In order to truly respect donor autonomy, the argument needs to go, it is not enough to allow them to provide and withdraw their broad consent to research. Dynamic consent is morally superior to broad consent because, by enabling study-specific consent, it truly respects the individual autonomy of donors.^3 4^

It certainly is true that dynamic consent would increase the number of occasions on which donors can make decisions. But this is not enough to justify the claim that it would truly respect individual autonomy. The requirement to seek consent from donors before using their biospecimen or health data for research gives moral space to the appreciable reasons that people may have not to contribute, ranging from concerns about the re-identification risks and possibility of incidental findings to a lack of trust in corporations or medical research at large. But dynamic consent goes no way in appreciating or helping to better understand these reasons. Instead, the model is built around the idea that it does not matter why people choose this way or another. Dynamic consent is futile as a way of respecting individual autonomy because it aims to enable not autonomous choice but mere choice.

Consider a fictional example. Melvin agrees that his genetic data may be included in a research platform enabling certain kinds of research into the genetic basis of diseases. The platform uses a dynamic consent approach to allow donors to be notified of, and give or withhold their consent to, each individual research study. Melvin agrees to provide his data for a study *A* but rejects the use of his data for another study *B*. Melvin may have a myriad of different reasons for deciding this way. But suppose that we come to find out that Melvin decided on a whim, without any particular reason altogether; or that he only accidentally pressed the ‘Reject’ button without noticing; or suppose that Melvin was acting on an unconscious gender bias (judging from the names, it seemed that the principal investigator behind *A* was a man, the principal investigator behind *B* a woman). In all of these cases, there is a concern with calling Melvin’s choice ‘autonomous’. Individual autonomy does not just mean the fact that people choose things, nor does respecting their autonomy mean to act on whatever they happen to choose. Autonomous choices are a certain species of choices.

This point has been forcefully argued by O’Neill.^17 31^ According to O’Neill, notions of autonomy in contemporary medical ethics have ‘lost touch with their Kantian origins, in which the links between autonomy and respect for persons are well argued’ (p. 5).^17^ Most such accounts require nothing more than for an autonomous choice to be ‘independent’ in some sense. Someone’s choice is autonomous so long as it can be considered their own, as opposed to someone else’s choice. But, as O’Neill notes, independent choices may well be ‘self-centred, pig-headed, impulsive, random, ignorant, out of control and regrettable or unacceptable for these and many other reasons’ (p. 28).^31^ Richer, philosophical accounts therefore understand autonomy as the capacity to make certain kinds of choices—choices that are self-assertive, self-knowing or critically reflective,^17^ associated with the capacity to form higher-order desires or evaluative judgements regarding the things that we want and that move us to act.^32 33^

Of course, O’Neill raises a problem here not just for dynamic consent but for the doctrine of informed consent generally.^34^ Informed consent, as understood in contemporary medical ethics, protects choices that are ‘bursting with individual autonomy’ just like it protects choices that are lacking in individual autonomy (p. 5).^17^ But one lesson to learn from this for the present discussion is that not all choices are equally worthy of respect. If autonomous choices are worthy of respect, then this cannot just be because they are choices but because they are expressions of a certain kind of self-reflective self-governance. For instance, respect for autonomy does not require that we act on the arbitrary, random, inconsiderate or morally objectionable choices of others. If this is so, then it remains unclear why we should think that the dynamic consent model, which asks us to do precisely this, would be especially well suited to satisfy the principle of respect for autonomy.

Proponents of dynamic consent could of course insist that dynamic consent would still be an improvement over broad consent. After all, even if dynamic consent cannot be said to enable autonomous choices in particular, it would also enable autonomous choices (alongside the odd ill-considered choice). Some additional options introduced by dynamic consent would be autonomy-enhancing. And this alone should be an improvement because these are options that people did not previously have under broad consent. Consider, for instance, that some donors might wish to specifically support female researchers or research into neglected areas, such as rare diseases or women’s health. Other donors might want to limit research with their genetic data to narrowly defined purposes because they belong to minority groups that have experienced genetic discrimination in the past. In general, the possibility of non-autonomous choice does not exclude the possibility of meaningful, self-reflected choice. And if donors can now make the latter kind of choices through a dynamic consent interface, certainly this must be a good thing.

However, while it certainly is a valuable aim to better account for the appreciable reasons that donors might have to limit the use of their biospecimen and data in research, there are three reasons to be sceptical that dynamic consent is the right way to promote this aim. First, there is justified scepticism that dynamic consent would actually provide much room for the kind of appreciable consent choices mentioned above. As practical examples show, dynamic consent will usually be implemented in a certain, narrower context, not for national or transnational biospecimen or health data research at large.^21–23^ In this regard, there is reasonable doubt that the kind of choices made possible through dynamic consent would often go beyond the trivial choice between studies that differ only in minor details.^9^ Against this background, the claim that dynamic consent would help donors become ‘equal partners’ in research seem, at best, to greatly exaggerate the meaningfulness of options introduced by the dynamic consent model.

Second, it is a mistake to think that every current problem of medical research, from traditional biases towards common diseases or men’s health to histories of science-driven discrimination, should and can be addressed through the function of consent. Informed consent ensures that the personal interests of the consent-giver are being accounted for. But where these interests are moral in nature, reliance on consent is not just unhelpful but out of place. For one thing, reliance on consent would imply that it is a matter of individual choice if we account for the traditional biases of medical research. Challenging the social biases and systemic injustices built into medical research is an important moral aim, regardless of whether a critical number of research participants want this. For another thing, it is an aim that cannot possibly be addressed through the means of consent. It is not our biospecimen or data that we think should be used towards this aim, everyone’s should. And our interest in contributing to this aim is not appropriately described by saying that we would want our biospecimen or data to be used in a certain way. Instead, we want medical research at large to take a certain direction; and this is where the principle of informed consent, which gives us authority only over our own affairs, reaches its limits.

And third, the problem remains that the dynamic consent model accounts for appreciable consent choices merely as a side effect of rubber-stamping all study-specific consent choices, regardless of their nature or appreciability. Dynamic consent treats self-reflective, positive, appreciable or morally praiseworthy choices on a par with random, unreflective or morally objectionable choices. In fact, proponents of dynamic consent are committed to the view that it does not even matter whether Melvin’s choice to limit the use of his data to study *A* was based on an unconscious gender bias or if he assertively did not want female researchers to be able to work with his data. Dynamic consent implies that his choice must be respected merely because it is his choice. But this is false. There is no moral obligation to limit the use of Melvin’s data to studies led by male researchers, even if this is what he wants. In the broad consent model, donors might not be able to personalise their consent any further; but this also means that they cannot discriminate between studies for trivial or objectionable reasons. There is an important conversation to be had about the additional consent choices that could be introduced to account for the appreciable reasons people might have for wanting to personalise their consent. But dynamic consent simply aims to bypass this conversation, as if it did not matter why people chose this way or another.

To be clear, we are not arguing that people would in fact frequently make ill-considered or morally problematic consent choices, though it seems unreasonable to us not to expect these choices to be statistically relevant. For instance, many studies have revealed a persistent gender bias in people’s dominant representations of scientific research and those carrying it out.^35–37^ How could it be rational to expect that a bias so deeply engrained in society would not have a statistically measurable impact on people’s consent choices? But ultimately the question of how statistically relevant these consent choices might be is an empirical one beyond the scope of our analysis. Our concern is rather with the normative implications of dynamic consent. The dynamic consent model implies that it does not matter whether people’s choices are appreciable, understandable and autonomous, or whether they are entirely arbitrary, random, expressions of harmful social biases or grounded in discriminatory attitudes. This strikes us as an unacceptable normative implication.

## Conclusion

While usually promoted as a way to enable truly autonomous consent choices in biobank and data infrastructure research, it is not obvious that the dynamic consent model would be morally superior to broad consent as a way to respect donor autonomy. For one thing, the idea that donors would be better informed under dynamic consent cannot justify a study-specific consent. For another thing, while dynamic consent increases the occasions on which donors can make decisions, it is neither obvious that this would introduce options for choice that meaningfully enhance donors’ autonomy, nor does it seem morally appropriate to act on every study-specific consent choice. It is no coincidence that the nature and structure of biobank and data infrastructure research have ‘forced a rethink of study-specific consent’.^38^ In the broad consent model, donors get to choose whether they want to provide their biospecimen and health data for certain kinds of medical research; they can withdraw consent at any time; additional ethical review and control measures should be in place to promote ethical research; and while the information materials in the consent process can be kept relatively lean, additional information can be made available in an easily accessible format on separate sites. If done well, it seems that broad consent is defensible and preferable to dynamic, study-specific consent.

Certainly, the conversation does not end here. We take it that part of what seems initially appealing about the dynamic consent model is that it can be understood as an extension of the widely shared view that contributions to medical research are entirely voluntary. Some might say that, in order to reflect the voluntary nature of research contributions, it is not enough to allow people to say ‘yes’ or ‘no’ to kinds of research. Instead, they need to be able to say specifically to which studies they wish to contribute. However, even when it comes to voluntary contributions to the general welfare, there usually are things that we can reasonably expect from one another. For example, medical ethicists have long debated the appropriateness of an opt-out model for postmortem organ donations, with some arguing that, in order to increase the supply of organs needed for transplantation, it is morally acceptable to assume people’s consent for donation unless they have explicitly refused.^39 40^ Some of course disagree. But the relevant point here is that expecting people to explicitly refuse rather than explicitly consent is, other things being equal, not thought to entail the view that organ donations are morally obligatory. Instead, the idea is that, given the great value for individual and public health, it can reasonably be expected of people who prefer not to donate to take action and communicate their refusal.

Currently, opt-out models are beginning to receive greater attention as a possible approach also to consent for biobank and data infrastructure research.^41^ In the European context, the planned European Health Data Space is a milestone here, making provisions for an opt-out model for the reuse of health data for medical research purposes.^42–44^ This strikes us as a step in the right direction. If broad consent for biobank and data infrastructure research is defensible and preferable to dynamic, study-specific consent, as we have argued, then it could also be possible to justify an opt-out model. Medical research creates significant benefits for individual and public health and those who wish not to contribute are free to refuse. An opt-out model would not seem to undermine the voluntary nature of research contributions. Rather, it would express the view that, given the value and benefits of medical research, it can reasonably be expected of people who do not want to contribute to explicitly refuse.

## Data Availability

No data are available.
